# Randomized controlled trial of pulse methyl prednisolone × placebo in treatment of pulmonary involvement associated with severe leptospirosis. [ISRCTN74625030]

**DOI:** 10.1186/1471-2334-11-186

**Published:** 2011-06-30

**Authors:** Ana Flávia C Azevedo, Demócrito de B Miranda-Filho, Gustavo T Henriques-Filho, Alfredo Leite, Ricardo AA Ximenes

**Affiliations:** 1 Department of Tropical Medicine, Federal University of Pernambuco, Recife, Brazil; 2 Department of Internal Medicine, School of Medicine Sciences, University of Pernambuco, Recife, Brazil

## Abstract

**Background:**

The lungs are involved in up to 70% of cases of leptospirosis. In the more severe forms-bleeding from the lungs and acute respiratory distress syndrome-the lethality is high. The treatment proposed for leptospirotic pneumonitis includes just care for patients in critical condition. Clinical and experimental studies point to the involvement of immunological mechanisms in the physiopathology of lung damage caused by leptospirosis. The aim of this study is to evaluate pulse treatment with methylprednisolone × placebo for leptospirotic pneumonitis.

**Study design:**

This is a randomized double-blind clinical trial to test the efficacy of pulse treatment with methylprednisolone in patients with leptospirotic pneumonitis, compared with a placebo. The patients are recruited from three hospitals in the city of Recife, in the Brazilian State of Pernambuco. The exclusion criteria include patients aged under 15 years, a history of hypersensitivity to the use of corticosteroids, the presence of active infection of fungal, tuberculous or bacterial origin apart from the infection by leptospira itself, the presence of hemoconcentration or atypical lymphocyte count on admission to hospital, the presence of co-morbidities that could be responsible for the radiological and gasometric alterations used to diagnose leptospirotic pneumonitis, evidence of recent cranial trauma, neurosurgery, peptic ulcer, and participation in another clinical trial. The patients are followed until they are discharged from hospital or die. The intervention consists of endovenous pulse treatment with 1 g methylprednisolone for three consecutive days in the study group and a placebo in the control group. The primary end-point is mortality from leptospirotic pneumonitis. The secondary end-points are: evolution of lung disease; the occurrence of nosocomial respiratory infection; duration of mechanical ventilation; duration of intensive care unit (ICU) stay; duration of hospital stay; occurrence of other infection-related complications; other respiratory complications; and adverse effects of methylprednisolone. The study is designed to recruit 266 patients and has a statistical "power" of 80% to detect a 50% reduction in mortality.

**Discussion:**

Lung involvement in leptospirosis is a serious manifestation, with a high and variable mortality rate. There is still no specific clearly-established treatment. Well-designed studies are needed to pave the way towards development of such a treatment.

## Background

Leptospirosis is an acute febrile infectious disease, which is potentially seriously and found all over the world. It is caused by spirochetes of the genus *Leptospira *[1]. In Brazil, it is a serious public health problem [2,3]. Clinical manifestations of leptospirosis are variable, ranging from sub-clinical infection or anicteric and self-limited fever to severe and potentially lethal disease with massive pulmonary hemorrhage or acute adult respiratory distress syndrome (SARA) [3-5]. The lungs are involved in leptospirosis in 16 to 70% of cases in different series and the frequency of such involvement has been on the rise and is becoming the main cause of death from the disease, in Brazil and in other countries [5,6]. Mortality rates for severe lung involvement may be as high as 50% [7]. Current treatment for leptospirotic pneumonitis is restricted to the basic care provided for patients in a critical condition [7,8]. The pathogenesis of such involvement has not yet been clearly established, although knowledge of the physiopathology of leptospirotic pneumonitis suggests the participation of immunological mechanisms in the development of pulmonary forms of the disease [9,10]. Two principal mechanisms have been suggested: vasculitis mediated by toxins and an exaggerated immune response in the host [11-14]. On the basis of evidence that immunological mechanisms are involved in the pathogenesis of lung involvement, high doses or a pulse of dexametasone or methylprednisolone have been used in patients with severe lung involvement in leptospirosis [15-17]. The aim of this article is to describe the design and methods to be used for a randomized double-blind clinical trial to evaluate the use of pulse treatment with methylprednisolone compared with a placebo in patients with lung involvement in letptospirosis.

## Methods/Design

### Study design

The study is a double-blind randomized clinical trial - RCT--which evaluates the efficacy of the use of pulse therapy with methylprednisolone compared to a placebo in patients with lung involvement in leptospirosis. The patients are randomly divided into two clearly-defined groups: a) the study group, which receives conventional treatment associated with endovenous pulse therapy with 1 g methylprednisolone for three consecutive days and b) the control group, which receives the conventional treatment plus a placebo. The patients are followed until such a time as they are discharged from hospital or die. The primary endpoint is taken to be mortality from leptospirotic pneumonitis. The secondary end-points are: a) evolution of lung disease; b) the occurrence of nosocomial respiratory infection; c) duration of mechanical ventilation; d) duration of intensive care unit (ICU) stay; e); duration of hospital stay; f) occurrence of other infection-related complications; h) Other respiratory complications; and i) adverse effects of methylprednisolone.

### Definition of Terms

#### Diagnosis of Leptospirosis

Patients are diagnosed as having leptospirosis when they present epidemiological information relating to contact with flooded areas or professional exposure; clinical criteria include the sudden onset of fever and myalgia; and three of the following laboratory criteria: normal leukometry or leukocytosis, thrombocytopenia, hyperbilirubinemia, increased muscle enzymes and the absence of hyperkalemia. Diagnosis of leptospirosis is confirmed by a serological test (antibody count using the ELISA method) collected after the seventh day and repeated after the tenth day if necessary.

#### Diagnosis of Leptospirotic pneumonitis

Diagnosis of leptospirotic pneumonitis is based on the presence of pulmonary interstitial infiltrate or bilateral alveolar-interstitial infiltrate on chest x-ray, in association with at least one of the following findings: alterations found by lung auscultation (fine crackles), cough, dyspnea, drop in hemoglobin, hemoptoic sputum, hemoptysis and hypoxemia, the latter being defined as a partial pressure of oxygen (PaO_2_) of less than 60 mmHg in arterial gasometry or peripheral saturation of hemoglobin with oxygen (SpO_2_) of less than 90% on pulse oximetry, both under ambient conditions, or an oxygenation índex (relation between PaO_2_/FiO_2_) of <300 mmHg.

### Definition of Variables

#### Independent Variable

The main intervention was the exposure to corticosteroid.

#### Dependent Variables

The dependent variables used to evaluate the primary and secondary outcomes are listed below in Table 1.

**Table 1:** Dependents variables to evaluate the primary and secondary outcomes.

Primary outcome	Mortality
Secondary outcomes	Evolution of lung disease
	Nosocomial respiratory infection
	Duration of mechanical ventilation
	Duration of intensive care unit stay
	Duration of hospital stay
	Occurrence of other infection-related complications
	Other respiratory complications
	Adverse effects of methylprednisolone

#### Mortality

The mortality outcome is defined as death caused leptospirosis related or not to leptospirotic pneumonitis.

#### Evolution of lung disease

The evolution of leptospirotic pneumonitis is evaluated by way of lung auscultation, radiological evaluation, gasometric evaluation and hematometry (hematocrit and hemoglobin) on a daily basis up to the seventh day and then on the 14th and 28th days. Respiratory auscultation is used to determine the presence of crackles. Once these have been identified, their severity is classified according to the extent of lung involvement (Table 2).

**Table 2:** Extent of lung involvement on respiratory auscultation

Severity of lung involvement	Extent of lung involvement on respiratory auscultation
Slight	Lower third
Moderate	Lower two thirds
Severe	Whole lung

A lung auscultation is carried out again on the fifth day of the patient's follow-up and categorized according to whether it is improving or worsening. It is considered to be improving when there is a reduction in the parameters described above and considered to be worsening if there is no change or if there has been an increase in the parameters.

The radiological evaluation aims to determine the presence and the extent of pulmonary infiltrate. When present, it is categorized according to its extent and severity (Table 3).

**Table 3:** Extent of lung involvement according to radiological examination

Severity of lung involvment	Extent of lung involvement according to radiological examination
Slight	Lower third
Moderate	Lower two thirds
Severe	Whole lung

Another radiological examination is carried out on the fifth day of the patient's follow-up and categorized as improving or worsening. It is considered to be improving when there is a decline in the radiological parameters described above. It is considered to have worsened when the parameters have remained unchanged or seen an increase.

In the gasometric evaluation the relation between PaO2/FiO2 is used in order to establish whether there has been an improvement or a decline. It is measured again on the fifth day and categorized as having improved when the value is equal to or greater than 300 and as having declined when the value is less than 300.

The evolution of hematometry is measured by way of hematocrit and hemoglobin levels and considered when there is a decrease of over three percentage points in hematocrit and 1 g/dl in hemoglobin, with no evidence of blood loss. The intensity of bleeding from the lungs is categorized as slight, when the hematocrit loss is more than three and less than five percentage points, moderate when greater than five but less than ten percentage points, and severe when it is greater than ten percentage points. The evolution of hematometry is evaluated on the fifth day of the patient's follow-up and is categorized as having improved when there has been no drop in hematometry or when it is kept in levels considered acceptable, when blood transfusion is necessary. It is considered to have declined when the hematometry remains unchanged after hemotransfusion or when there is an increased drop in hematometry.

Patients are considered to have improved, irrespective of the extent of the improvement, when, after five days of follow-up, there has been an improvement in three of the four parameters evaluated: lung auscultation, radiological evaluation of pulmonary infiltrate, arterial gasometry and hematometry. Improvement is considered to be early when it occurs within the first forty-eight hours and late when it occurs later than this. Treatment is considered to have failed when the patient does not show improvement in three of the four parameters listed above.

#### Respiratory Infection

Respiratory infection is defined by the presence of a recent pulmonary infiltrate or an increase in pre-existing infiltrate, in association with at least two or three clinical criteria: a) fever (axillary temperature of 37.8°C), b) changes in leukometry (leukocytes <4000 cel/mm3 or >11000 cel/mm3), c) purulent tracheo-bronchial secretion.

#### Duration of mechanical ventilation

The duration of mechanical ventilation outcome is evaluated according to whether the patient did or did not require ventilator assistance for a continuous number of days until fully taken off the ventilator.

#### Duration of intensive care unit (ICU) stay

The duration of ICU stay outcome is evaluated according to whether the patient did or did not stay a continuous number of days in the ICU before being definitively discharged from the ICU.

#### Duration of hospital stay

The duration of hospital stay outcome is evaluated according to whether or not the patient stay in hospital for a continuous number of days before being definitively discharged from hospital.

#### Occurrence of other infection-related complications

Occurrence of other infection-related complications may be present when the patient is diagnosed as having infection of the urinary tract, phlebitis, cellulitis, a pulmonary abscess, tracheostomy wound infection, sepsis, septic shock, or fungemia.

#### Other respiratory complications

The respiratory complications considered are atelectasis, pneumothorax, broncospasms, bronco-aspiration, pneumomediastinum, subcutaneous emphysema, hemothorax, decompensation of chronic obstructive pulmonary disease, acute pulmonary embolism, and empyema.

#### Adverse effects of methylprednisolone

Possible adverse effects of methylprednisolone include gastro-intestinal bleeding with a drop in hematometry, fungal infection, Herpes zoster, bacterial infection, hyperglycemia, and systemic arterial hypertension (TA > 149 × 90 mmHg).

### Population

Patients who fulfill the diagnostic criteria for leptospirotic pneumonitis are included. Patients are excluded if they are aged less than 15 years, have a history of hypersensitivity to corticosteroids, have an active infection of fungal, tuberculosis or bacterial origin other than the leptospira infection, have hemoconcentration or lymphocytic atypia on admission to hospital, have co-morbidities that may be responsible for changes in radiological and gasometric examinations used to diagnose leptospirotic pneumonitis, present evidence of recent cranial trauma, neurosurgery or a peptic ulcer, or if they are taking part in another clinical trial.

### Participant recruitment

The study will cover patients admitted to the intensive care unit, or to the infectious diseases or internal medicine ward in the Oswaldo Cruz University Hospital, Barão de Lucena Hospital and Clinics University Hospital. During the study, the recruitment of patients from other hospitals will be allowed.

### Ethical considerations

The study was approved by the Research Ethics Committee, according to the resolutions of the National Health Council (FR - 167386) and was monitored by an external data monitoring committee throughout.

### Sample size

The sample size was calculated on the basis of a statistical significance (alpha error) of 5%, a power of 80%, corresponding to a beta error of 20%. The expected mortality for leptospirotic pneumonitis is 30%. For a reduction of 50% in mortality, the expected mortality in patients treated with corticosteroids would be 15%. The sample size calculated was 133 patients for each group. The same parameters were used to obtain statistical significance and a standard deviation of 21 days for duration of mechanical ventilation, giving an expected sample of 33 patients per group. For the duration of ICU stay variable, the same parameters were used to obtain statistical significance and a standard deviation of 8 days, giving an expected sample of 23 patients per group.

### Randomization

The patients with clinical symptoms of leptospirosis admitted to ICUs and hospital wards covered by the study are evaluated by doctors involved in the study to establish the extent to which they meet the criteria for diagnosis of leptospirosis and leptospirotic pneumonitis. Once this has been established, the patient is informed of the study and inclusion depends on his or her consent (signed informed consent), or, if this is not possible, on that of their relatives or legal guardians. The randomization was carried out in advance by drawing lots. The groups to which the patients are allocated are placed in sealed numbered envelopes that are only opened after the informed consents have been signed. The envelopes are opened by a third party not involved in assistance of the patients. Block randomization within each severity stratum is performed in each hospital unit. The degree of severity is stratified according to the APACHE II score. Blocks of ten are used.

### Operationalization of the Study

The study involves three hospitals and their respective intensive care units and wards for internal medicine and infectious diseases. A coordinating physician is assigned to each hospital to organize evaluation, inclusion and follow-up of the patients for the duration of the study. Two supervising doctors oversee the development of the study by way of monthly meetings with the coordinators of each hospital. To ensure that the study is double-blind, the preparation of the methylprednisolone and of the placebo is carried out in each hospital's pharmacy, duly coded and known only to the nurse involved in the study. The patients in the control group receive an infusion of saline solution identical in volume to that of the dilution received by the intervention group. The nurse involved in the study fill in a card identifying which patients received the placebo and which the methylprednisolone. This card is stored by a physician who did not treat the patients nor takes part in the investigation and will only be opened at the end of the study.

### Standardization of Treatments

All the patients included receive the conventional treatment for leptospirosis, which is the basic care provided for severely ill patients, such as volemic reposition, antibiotics, control of hemodynamics, correction of electrolytic and acid-base disorders, transfusion of hemoderivatives, hemodialysis, early and adequate nutrition, oxygen treatment, and artificial ventilation, when necessary, along with others that are commonly recommended. Decisions regarding the use of drugs and invasive or non-invasive procedures are taken by the patients' attending physicians, in accordance with current good medical practice, based on data from the literature. In view of the high doses of corticosteroid used, all patients are given an anti-parasitic drug (albendazol) and gastric protection (H2 blocker). The specific items of the treatment of leptspirosis are standardized as follows:

• Volemic reposition and the use of vasoactive drugs for lungs congested by pneumonitis follow the criteria established in the algorithms given in Appendices II and III.

• Treatment with antibiotics involves the administration of crystalline penicillin at a dose of 1,500,000 international units every six hours for all patients, regardless how long they have had the disease, or with a 1 g dose of ceftriaxone every twenty-four hours in those allergic to penicillin or where the use of penicillin is not possible. Antimicrobial treatment for associated infections is carried out according to specific recommendations^13,14,31^.

• The recommendation for platelet transfusion is calculated on the basis of one unit of platelet concentrate per 10 kg of the patient's weight, every twelve or eight hours, according to the following criteria: a platelet count (PC) less than or equal to 20,000/mm^3 ^even in the absence of bleeding; a PC of less than or equal to 50,000/mm^3 ^in cases of more severe bleeding and/or when invasive surgery or biopsies are required^15 ^and PC <100,000/mm^3 ^in cases where major surgery has been performed.

• Hemodialysis is carried out according to the recommendations outlined in the algorithms contained in Appendices II and III.

• Mechanical ventilation, where necessary, is used in controlled volume mode (VCV with a flow rate of 6 ml/kg, to generate a maximum plateau pressure of 30 cmH_2_O and a maximum inspiratory peak of 45 cmH_2_O) or controlled pressure (PCV with maximum pressure of 30 cmH_2_O), and with final positive expiratory pressure sufficient to maintain the lowest possible fraction of inspired oxygen (FiO_2_) (the ideal PEEP calculated according to the routine of the hospital ubit: PEEP curve X static complacency; pressure curve X volume; or a test of different PEEPs under SpO_2 _monitoring)^2,3,18,42^.

### Statistical analysis

Double data enter is performed using EPI INFO Version 6.0, giving rise to two datasets that can be compared to detect possible typing errors. The dataset is subsequently checked for consistency. The distribution of potential confounding factors in the two treatment groups are compared to check randomization.

The main analysis will be carried out through comparison of the various outcomes in the two groups, by calculating the percentage distribution and the relative risk. The 95-% confidence interval and the chi-squared test will be used to test statistical association. The t-test will be used to compare the means. The results are considered statistically significant when the p-value is less than 0.05. The efficacy of the intervention is calculated according to the following formula:

Patients randomly included in the study who, for whatever reason, including transfer to another hospital, do not receive the treatment for which they were assigned are considered to belong to the group to which they were allocated **(intention to treat analysis)**. These will be deemed treatment failure. The patients who do not receive confirmation from the laboratory of diagnosis of leptospirosis will still be included in the analysis. They will not be considered as losses or treatment failure. Data analysis will be carried out using EPI INFO Version 6.0 and SPSS 8.0.

## Discussion

Lung involvement in leptospirosis is a serious manifestation, with a high and variable mortality rate. There is still no specific cleary-established treatment. Well-designed studies are needed to pave the way towards development of such a treatment.

## Authors' contributions

All authors contributed to study design and mentoring, to the conceptualization of the intervention, to the recruitment plan and to the implementation of the study. All authors read and approved the final manuscript.

## Pre-publication history

The pre-publication history for this paper can be accessed here:

http://www.biomedcentral.com/1471-2334/11/186/prepub
